# Supplementary material for: MetaRibo-Seq measures translation in microbiomes
Source: Nat Commun. 2020 Jun 29;11:3268. doi: 10.1038/s41467-020-17081-z (PMC7324362; doi:10.1038/s41467-020-17081-z)
Supplement: Supplementary file 10 — Supplementary Data 7 [file 41467_2020_17081_MOESM10_ESM.zip › File2/Confidence_High_Taxonomy/41396_out.krona.html]

Javascript must be enabled to view this page.

members
magnitude
magnitudeUnassigned
count
unassigned
taxon
rank

41396\_out

48

superkingdom
48
2

48
976
phylum

200643
48
class

order
171549
48

171552
48
family

genus
838
48


SRS144537\_contig\_number\_41515
species
1
1262925

165179
36
species

SRS043667\_contig\_number\_1038SRS043841\_contig\_number\_8059SRS044535\_contig\_number\_11512SRS045713\_contig\_number\_21745SRS046502\_contig\_number\_contig-100\_227.88448SRS047433\_contig\_number\_13719SRS049896\_contig\_number\_9601SRS049995\_contig\_number\_13351SRS050752\_contig\_number\_11879SRS053356\_contig\_number\_40335SRS053398\_contig\_number\_14698SRS054059\_contig\_number\_332SRS058145\_contig\_number\_contig-100\_16784.16785SRS064757\_contig\_number\_20053SRS065397\_contig\_number\_5604SRS074586\_contig\_number\_1243SRS077194\_contig\_number\_355SRS077392\_contig\_number\_contig-100\_24118.56752SRS077849\_contig\_number\_29418SRS077991\_contig\_number\_18SRS078176\_contig\_number\_337SRS078419\_contig\_number\_17048SRS103987\_contig\_number\_3702SRS104036\_contig\_number\_6493SRS1041031\_contig\_number\_26683SRS1041116\_contig\_number\_6869SRS1041140\_contig\_number\_19147SRS104327\_contig\_number\_1060SRS105082\_contig\_number\_9045SRS143181\_contig\_number\_386SRS143876\_contig\_number\_16269SRS144603\_contig\_number\_13721SRS146764\_contig\_number\_19419SRS147614\_contig\_number\_5135SRS148253\_contig\_number\_10610SRS150029\_contig\_number\_14636


SRS047044\_contig\_number\_8866SRS049712\_contig\_number\_13095SRS097958\_contig\_number\_13512SRS100907\_contig\_number\_contig-100\_27267.27268SRS1041134\_contig\_number\_8582SRS1041137\_contig\_number\_28804SRS144297\_contig\_number\_7795SRS144506\_contig\_number\_48662SRS144714\_contig\_number\_21701
species
59823
9

1262937
1
species

SRS077641\_contig\_number\_16969

1
1262926
species

SRS104912\_contig\_number\_13524
